# Neuroprotection by Curcumin in Ischemic Brain Injury Involves the Akt/Nrf2 Pathway

**DOI:** 10.1371/journal.pone.0059843

**Published:** 2013-03-28

**Authors:** Jingxian Wu, Qiong Li, Xiaoyan Wang, Shanshan Yu, Lan Li, Xuemei Wu, Yanlin Chen, Jing Zhao, Yong Zhao

**Affiliations:** 1 Department of Pathology, Chongqing Medical University, Chongqing, People’s Republic of China; 2 Department of Pathophysiology, Chongqing Medical University, Chongqing, People’s Republic of China; Massachusetts General Hospital/Harvard Medical School, United States of America

## Abstract

Oxidative damage plays a critical role in many diseases of the central nervous system. This study was conducted to determine the molecular mechanisms involved in the putative anti-oxidative effects of curcumin against experimental stroke. Oxygen and glucose deprivation/reoxygenation (OGD/R) was used to mimic ischemic insult in primary cultured cortical neurons. A rapid increase in the intracellular expression of NAD(P)H: quinone oxidoreductase1 (NQO1) induced by OGD was counteracted by curcumin post-treatment, which paralleled attenuated cell injury. The reduction of phosphorylation Akt induced by OGD was restored by curcumin. Consequently, NQO1 expression and the binding activity of nuclear factor-erythroid 2-related factor 2 (Nrf2) to antioxidant response element (ARE) were increased. LY294002 blocked the increase in phospho-Akt evoked by curcumin and abolished the associated protective effect. Adult male Sprague-Dawley rats were subjected to transient middle cerebral artery occlusion for 60 minutes. Curcumin administration significantly reduced infarct size. Curcumin also markedly reduced oxidative stress levels in middle cerebral artery occlusion (MCAO) rats; hence, these effects were all suppressed by LY294002. Taken together, these findings provide evidence that curcumin protects neurons against ischemic injury, and this neuroprotective effect involves the Akt/Nrf2 pathway. In addition, Nrf2 is involved in the neuroprotective effects of curcumin against oxidative damage.

## Introduction

Reactive oxygen species (ROS) generated by disturbances of the oxidation/reduction state of the cell have been implicated in the pathogenesis of various vascular diseases, cancers and neurodegenerative disorders. Therefore, the intervention of oxidative damage using compounds with antioxidant properties may relieve or prevent diseases in which oxidative stress is the primary cause [1]. Curcumin {1,7-bis(4-hydroxy-3-methoxyphenyl) -1,6-heptadiene-3,5-dione} (diferuloyl methane), the principle coloring agent present in the rhizomes of Curcuma longa (zingiberaceae), possesses many therapeutic properties including anti-oxidant [2], anti-inflammatory [3] and anti-cancer properties [4]. Several studies have indicated that curcumin has protective effects against cerebral ischemia in rats and gerbils [5]–[7]. In our previous studies, we demonstrated that curcumin could significantly reduce the volume of brain infarcts and neurological dysfunctions that follow transient middle cerebral artery occlusion (MCAO) in rats [8], [9].

The Phosphatidylinositol 3-kinase(PI3K)/Akt pathway has been shown to play a crucial role in the mechanisms promoting cell survival, which are driven by growth factors [10]. Recent evidence indicates that this pathway is capable of maintaining and/or enhancing the survival of neurons [11], [12]. Furthermore, the Akt phosphorylation facilitated the translocation of nuclear factor-erythroid 2-related factor 2(Nrf2), its downstream transcription factor, to the nucleus which could induce expression of genes encoding phase II drug-metabolizing enzymes such as NAD(P)H:quinone oxidoreductase1(NQO1) [13], [14], glutathione S-transferase (GST) [15], aldoketo- reductase(AR) [16], hemeoxygenase-1(HO-1) [17], and so on. The induction of the phase II enzyme system can eliminate or inactivate a diverse array of electrophilic and oxidative toxicants before they cause damage to critical cellular macromolecules. Kang et al. [18] reported that curcumin up-regulates AR expression via Nrf2 in a PI3K/Akt-dependent manner against oxidative stress damage in vascular smooth muscle cells (VSMC). Considering the key role of PI3K/Akt in cell survival in neurotoxicity models, we sought to determine whether PI3K/Akt is involved in the neuroprotective effect of curcumin. Oxygen and glucose deprivation (OGD) in primary cultured neurons was used to mimic ischemic insult in vitro. In addition, transient ischemia with reperfusion 1 h after stroke onset was used to assess neuroprotection of curcumin in vivo. We demonstrated that curcumin, a known antioxidant, reduced oxidative stress generated by ischemia/reperfusion (I/R) and promoted cell survival involving Akt/Nrf2 signal pathway.

## Materials and Methods

### Animals and Reagents

We used nine-week-old Sprague-Dawley male rats (250±20 g) in in vivo study. Newborn Sprague-Dawley rats (days 0–1) were obtained to culture primary cortical neurons. All experiments were approved by the institutional Animal Care and Use Committee of the Chongqing Medical University. Curcumin was purchased from Sigma. General reagents were obtained from Sigma-Aldrich (St Louis, MO, USA), unless stated otherwise.

### Primary Culture of Rat Cortical Neurons

Cortical neurons were prepared from brains of one-day-old Sprague-Dawley rats as previously described [19], [20] . Approximately 30,000 cells in 50 µl neurobasal medium containing glutamine (1 mmol/L), 1% penicillin, streptomycin (Pen/Strep), and 10% fetal bovine serum were seeded into 6-well plates. After 2 h, 0.5 ml neurobasal medium containing the serum-free B27 supplement (2%), Pen/Strep, and glutamine were added to each well. After 2 days in vitro (DIV), 5 µM cytosine arabinofuranoside was added to inhibit neuronal proliferation. At 5 DIV, the medium was changed to fresh neurobasal medium containing B27, Pen/Strep, and glutamine. Neurons were maintained in a humidified incubator with 5% CO_2_/balance air (result: 20% O_2_). Glial growth was suppressed by addition of 5-fluoro-2-deoxyuridine and uridine, yielding cultured cells with 90% neurons as determined by NeuN and glial fibrillary acidic protein (GFAP) staining. The medium were replaced with fresh medium every 3 days. Experiments were performed on days 7–10. The experiments were conducted under a protocol approved by the Institutional Animal Care and Use Committee of Chongqing Medical University.

### Oxygen-glucose Deprivation

Rat cortical neurons were deprived of O_2_ and glucose by changing the culture medium to a glucose-free “ionic shift” solution (ISS) with a pH 6.55, containing NaCl (39 mM), Na-gluconate (11 mM), K-gluconate (65 mM), NMDG-Cl (38 mM), NaH_2_PO_4_ (1 mM), CaCl_2_ (0.13 mM), and MgCl_2_ (1.5 mM), Bis-Tris (10.5 mM) as previously described (Ming et al. 2006). The coverslips were placed in an anaerobic chamber (Thermo Forma Scientific3131) in an atmosphere of 10% H2, 5% CO_2_ and 85% N_2_. The O_2_ and H_2_ concentrations inside the chamber were monitored by using a Monitor Analyzer (Coy Laboratory Products, Inc) and values of <1 part O_2_ per million and H2 between 5–6% were considered acceptable. The dissolved O_2_ concentration of the ISS solution was measured using the CHEMet test (CHEMetrics, VA, USA) that employs the Rhodazine D method. The values of the dissolved oxygen in deoxygenated ISS were between 10 and 40 parts per billion, which is equivalent to 0.32–1.28 µM. After washing the cells twice with deoxygenated ISS, they were incubated in the anaerobic chamber for 1 h. Subsequently, the cells were removed from the anaerobic environment, the ISS was replaced with serum free medium (DMEM/F12) containing 5 mM glucose, and cultures were placed in an incubator with 95% air (20% O_2_)/5% CO_2_. Control experiments were performed with cells maintained under identical conditions before, during, and after OGD except that during the sham OGD they were maintained in serum free medium that contained 5 mM glucose. Control and treated cells were treated identically except that they were not exposed to OGD. To observe the effect of curcumin post-treatment, cortical neurons were post-treated with various concentrations of curcumin (2.5, 5.0, 10 and 25 µM) after a 1-h period of OGD and subjected to OGD for 1 h followed by 24 h of reoxygenation. The solvent DMSO (maximum 0.1% final concentration) served as the control. Neurons were post-treated with signal inhibitors (all from Calbiochem, San Diego, CA, USA) for p38 MAPK (5 lg/mL of SB203580), ERK (5 lg/mL of PD98059), JNK (5 lg/mL of SP600125), and PI3K (10 lg/mL of LY294002 or Wortmannin) as previously described.

### Cell Viability Assay

The viability of cells was examined by 3-(4, 5-dimethylthiazole-2-yl)- 2,5-dipenyltetrazolium bromide (MTT) assay. After subjected to OGD for 1h, neurons were treated with 2.5, 5.0, 10 and 25 µM curcumin respectively and subjected to OGD for 1 h followed by 24 h of reoxygenation. MTT was added to a final concentration of 0.5 mg/ml for 4 h before the end of the experiment. The supernatant was removed and 150 µl DMSO was added for 20 min. The MTT optical density values were measured on a microplate reader at 570 nm and 630 nm wavelengths (BioRad).

### Cell Injury Assay

The cell injury was detected by measuring lactate dehydrogenase (LDH). After OGD 1 h, Neurons were incubated with 2.5, 5.0, 10 and 25 µM curcumin and subjected to OGD for 1 h followed by 24 h of reoxygenation. LDH release was measured in culture medium using the LDH assay kit (Roche Molecular Biochemicals). Medium (100 µl) was transferred from culture wells to 96-well plates and mixed with the 100 µl reaction solution provided in the kit. Optical density was measured at 492 nm 30 min later using a microplate reader (BioRad). Background absorbance at 620 nm was subtracted. The maximal releasable LDH was obtained in each well after 15 min incubation with 1% Triton X-100 at the end of each experiment.

### MCAO Model

One-hundred-thirty-six adult male Sprague-Dawley rats (250±20 g) were divided randomly into the following 4 groups: sham-operated, vehicle- treated I/R group (vehicle group), curcumin-treated group (cur group) and curcumin and LY294002- treated group (cur+ LY group)(n = 34 for each group). Rats were anesthetized with chloral hydrate (350 mg/kg i.p.) and subjected to MCAO as described in detail in our previous study [9], [21], [22]. In brief, we surgically exposed the left common carotid artery, internal carotid artery, and external carotid artery. A 4-0 monofilament nylon suture (Japan, Sunjos) with a rounded tip was inserted into the internal carotid artery through the external carotid artery stump and gently advanced to occlude the MCA. After 60 minutes of MCAO, the suture was removed to restore blood flow (reperfusion confirmed by laser Doppler). Sham-operated rats were manipulated in the same way, but the MCA was not occluded. Regional cerebral blood flow (rCBF) was monitored by laser-Doppler flowmeter (Periflux System 5010, Perimed Inc) with the use of a flexible probe over the skull. rCBF was measured before ischemia, during MCAO, and during reperfusion. Animals that did not show a CBF reduction of at least 70% and animals that died after ischemia induction were excluded from the experimental group. Core body temperature was monitored with a rectal probe and maintained at 37°C during the entire procedure. Mean arterial blood pressure (of the left femoral artery), pH, arterial blood gases, and blood glucose levels before, during, and after ischemia were measured. The total elimination rate was 18/136. The experiments were conducted under a protocol approved by the Institutional Animal Care and Use Committee of Chongqing Medical University.

### Administration of Drugs

Curcumin (Sigma) was dissolved in 5 N NaOH and titrated to pH 7.4 using 1 N HCl, and diluted with physiological saline to a concentration of 300 mg/kg, which was administered by intraperitoneal injection. To investigate the role of the PI3K/Akt pathway after MCAO, LY294002 (Sigma), a PI3K inhibitor, was dissolved in dimethyl sulfoxide and phosphate-buffered saline (PBS) and injected intracerebroventricularly (25 mmol/L, bregma; 1.0 mm lateral, 0.2 mm posterior, 3.1 mm deep). Vehicle of 2 ml/kg physiological saline (i.p.),300 mg/kg curcumin and 300 mg/kg curcumin combined with 2 µl LY294002 were administered 1 h after MCAO at reperfusion onset according to our previous studies [9].

### Measurement of Infarct Volume

Animals were killed 24 h after reperfusion and brains were rapidly removed and frozen at −20°C for 5 min. The brains (n = 8 for each group) were sliced into 2 mm thick coronal sections and immersed in 2% 2,3,5-tripenyltetrazolium chloride (TTC) at 37°C for 20 min. After the end of staining, color images of these slices were captured using a digital camera (Canon EOS400) and the software Adobe Photoshop 7.0. The size of infarct was calculated with the Mias-2000 image analysis system (Institute of Image and Graphics, Sichuan University, China). Infarcted areas of all sections were added to derive the total infarct area, which was multiplied by the thickness of the brain sections to obtain the infarct volume. To compensate for the effect of brain edema, the corrected infarct volume was calculated as follows: corrected infarct area = measured infarct area×{1-[(ipsilateral hemisphere area-contralateral hemisphere area)/contralateral hemisphere]} [23].

### Determination of Oxidative Stress

Left cortical samples (n = 6–7 for each group) were weighed. Malondialdehyde(MDA) level and superoxide dismutase (SOD) activity were measured as previously described [24]. In brief, MDA level was measured using the thiobarbituric acid method. The amount of lipid peroxide was measured as the production of MDA. Absorbance was measured at 532 nm by spectrometry. SOD activity was measured using the xanthine oxidase method. Absorbance was determined at 550 nm by spectrometry. MDA and SOD kits were purchased from the Nanjing Jiancheng Bioengineering Institute, Nanjing,China. All protein concentrations of cortical tissue homogenate samples were determined with the Coomassie blue method (assay kit was purchased from Bio-Rad).

### Western Blot Analysis

After 24 h of reperfusion, each rat was executed under 10% chloral hydrate anesthesia. Left cortical samples (n = 6–8 for each group) were weighed and protein was extracted. The cortical neurons were washed twice with ice-cold PBS. For the whole cellular lysate preparation, the samples were homogenized in an ice-cold buffer (tris-(hydroxymethyl)- aminomethane 50 mmol/L, pH 7.4, NaCl 150 mmol/L, 0.5% TritonX-100, edetic acid 1 mmol/L, phenylmethylsulfonyl fluoride 1 mol/L, and aprotinin 5 mg/L), and centrifuged at 14,000×g at 4°C for 30 min. Then, the supernatants were collected as total proteins. Proteins lysates were electrophoresed through a 15% sodium dodecyl sulfate polyacrylamide gel (SDS-PAGE), and electrically transferred to a nitrocellulose membrane. This membrane was overnight incubated at 4°C in tris-(hydroxymethyl)-aminomethane buffered saline (TBS) containing 5% milk, and detected with the primary rabbit polyclonal antibody against Akt, phospho-Akt (1∶1000 dilution, Cell Signaling), Nrf2, NQO1 (1∶500, Santa Cruz). After washing with TBST, membranes were incubated with the secondary antibodies (Santa Cruz, 1∶1000) for 1 h at room temperature. Western blots were developed with the ECL chemiluminescence system (GE Healthcare) and were captured on autoradiographic films (Kodak). Films were scanned and densitometric analysis of the bands was performed with Labworks 4.6 Analysis System (UVP.Inc, USA).

### Nuclear Extract Preparation and Electrophoretic Mobility Shift Assay (EMSA)

Nuclear extracts were prepared according to a previously published method [25]. Briefly, frozen brain samples were trypsinized and suspended in buffer A (10 mM HEPES, pH 7.9, 10 mM KCl, 0.1 mM EDTA, 0.1 mM EGTA, 1 mM DTT, 0.5 mM PMSF) (all from Sigma Chemical Co.). The homogenates were incubated on ice for 25 minutes and vortexed for 30 seconds after addition of 50 *µ*L 10% NP-40 (Sigma Chemical Co.). The mixture was then centrifuged for 10 minutes at 5000 g at 4°C. The pellet was suspended in 200 *µ*L ice-cold buffer B composed of 50 mmol/L HEPES (pH 7.9), 400 mM NaCl, 50 mmol/L KCl, 0.1 mmol/L EDTA, 1 mmol/L DTT, and 0.5 mmol/L PMSF, and 25% (v/v) glycerol and incubated on ice for 30 minutes with frequent mixing. After centrifugation (12,000 ×g, 4°C) for 15 minutes, the supernatants containing nuclear proteins were collected and stored at *−*80°C for further analysis.

EMSA was performed using the commercial Chemiluminescent EMSA kit (Pierce Biotechnology). For EMSA, 5 µg of total extracted nuclear proteins was incubated with 1 pmol double-stranded [*γ*-^32^P] ATP end-labeled oligonucleotide probe containing the sequences [26](sense strand, 5^/−^TTTTCTGCT-GAGTCAAGGTCCG- 3^/^; antisense strand, 3^/−^AAAAGACGACT-CAGTTCCAGGC-5^/^) in binding buffer (10 mM HEPES, pH 7.9, 80 mM NaCl, 3 mM MgCl2, 0.1 mM EDTA, 1 mM dithiothreitol, 1 mM phenylmethylsulfonyl fluoride, and 10% glycerol). After the incubation, the samples were loaded on a 5% TBE–polyacrylamide gel (Bio-Rad) and electrophoretically separated in 0.5X TBE buffer. The gel was vacuum-dried and exposed to X-ray film (Fuji Hyperfilm, Tokyo, Japan) at −80°C with an intensifying screen. Levels of Nrf2 DNA binding activity were quantified by computer-assisted densitometric analysis.

### Statistical Analysis

All data are expressed as Mean±SEM. Statistical differences between the various groups were assessed with a one-way ANOVA followed by a post hoc test. Comparisons between two groups were assessed using the unpaired *t* test. A value of *P*<0.05 was considered statistically significant.

## Results

### *C*urcumin Post-treatment Improves Survival of Cortical Neurons and Reduced Cell Injury Induced by OGD/R

**Figure 1:**
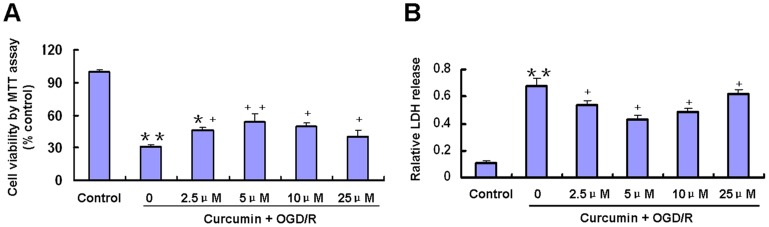
Effects of curcumin on primary cultured cortical neurons exposed to reoxygenation 24 h after OGD for 1 h. Curcumin at indicated concentrations applied to neurons 24 h after a 1 h period of OGD. A, Cell viability was measured by 3-(4, 5-dimethylthiazole-2-yl)-2,5-dipenyl- tetrazolium bromide assay. Data were normalized by control as 100% (n = 6) B, Cell injury was measured by lactate dehydrogenase release assay (n = 6) *p<0.05, **p<0.01 versus Control; +p<0.05, ++p<0.01 versus OGD/R without curcumin.

As shown in Figure 1A, MTT activity was significantly decreased by OGD/R treatment (p<0.01). Curcumin (2.5–25 µM) significantly increased cell viability reduced by OGD/R treatment (p<0.05; Figure 1A). Compared to the OGD/R group, the viability of the groups treated with 5 µM curcumin was increased by approximately 23% (p<0.01), while the viability of the groups treated with 2.5, 10 and 25 µM curcumin was increased by approximately 16%,18% and 10%, respectively (p<0.05).

LDH released in the incubation buffer in cells subjected to OGD and following 24 h reoxygenation was significantly higher than that in control cells (Figure 1B). Post-treatment of the cortical neurons with 2.5, 5.0, 10 and 25 µM curcumin immediately after OGD blocked the OGD/R-induced LDH release (Figure 1B). Post-treatment with 5 µM curcumin significantly reduced the relative LDH release to 43.2±3.1% compared with 68.4±5.2% in OGD/R cells (p<0.05). Combined with the results of MTT activity and LDH release, incubation control neurons with 5 µM curcumin post-treatment for 24 h in DMEM/F12 medium without serum shows better neuroprotection than other doses of 2.5, 10 and 25 µM curcumin (p<0.05). Therefore, a 5 µM concentration of curcumin was used in the following experiments.

### Suppression of the PI3K/Akt Pathway Antagonize Neuroprotective Effects of Curcumin

**Figure 2:**
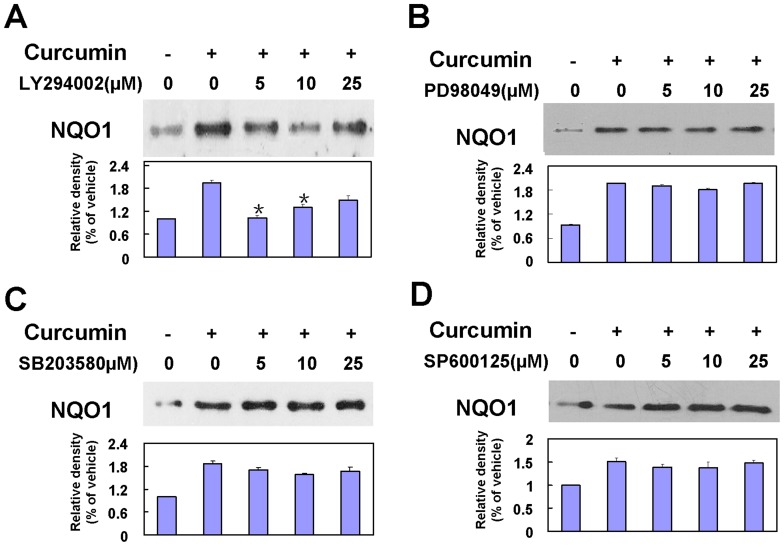
Effects of PI3K/Akt activation on curcumin-induced NQO1 expression. A–D: Effects of inhibitors of PI3K and MAPKs on curcumin-induced NQO1 protein expression. Cortical neurons were post-treated with various concentrations of (A) LY294002, (B) SB203580, (C) SP600125, or (D) PD98059 and were incubated with 5 µM curcumin for 24 h. Data were normalized by vehicle group as 100%. Bars represent the means±SE (n = 4–6), *p<0.05 compared with the curcumin-treated group.

Curcumin (5 µM) was added to cultures after the OGD insult, and at 24-h reoxygenation the total cellular protein was isolated and analyzed for NQO1 protein expression by immunoblot assay. To characterize the redox signaling pathway involving curcumin-mediated NQO1 induction, effects of specific inhibitors of the PI3K/Akt pathway and three MAPK cascades were examined. Cultures of cortical neurons were post-treated with curcumin in the presence or absence of signaling inhibitors for PI3-K (LY294002), JNK (SP600125), ERK1/2 (PD98059), and p38 MAPK (SB203580). Compared to the curcumin+OGD/R group, the curcumin-induced increase in NQO1 total protein levels was significantly suppressed by LY294002 at concentrations of 5 and 10 µM (p<0.05) (Figures 2A–2D).

### Physiological Data

No statistical differences were observed in the experimental groups with regard to mean arterial blood pressure, heart rate, arterial blood gases, glucose levels and rCBF before, during, or after ischemia (data not shown).

### Curcumin Attenuated Infarction Volumes

**Figure 3:**
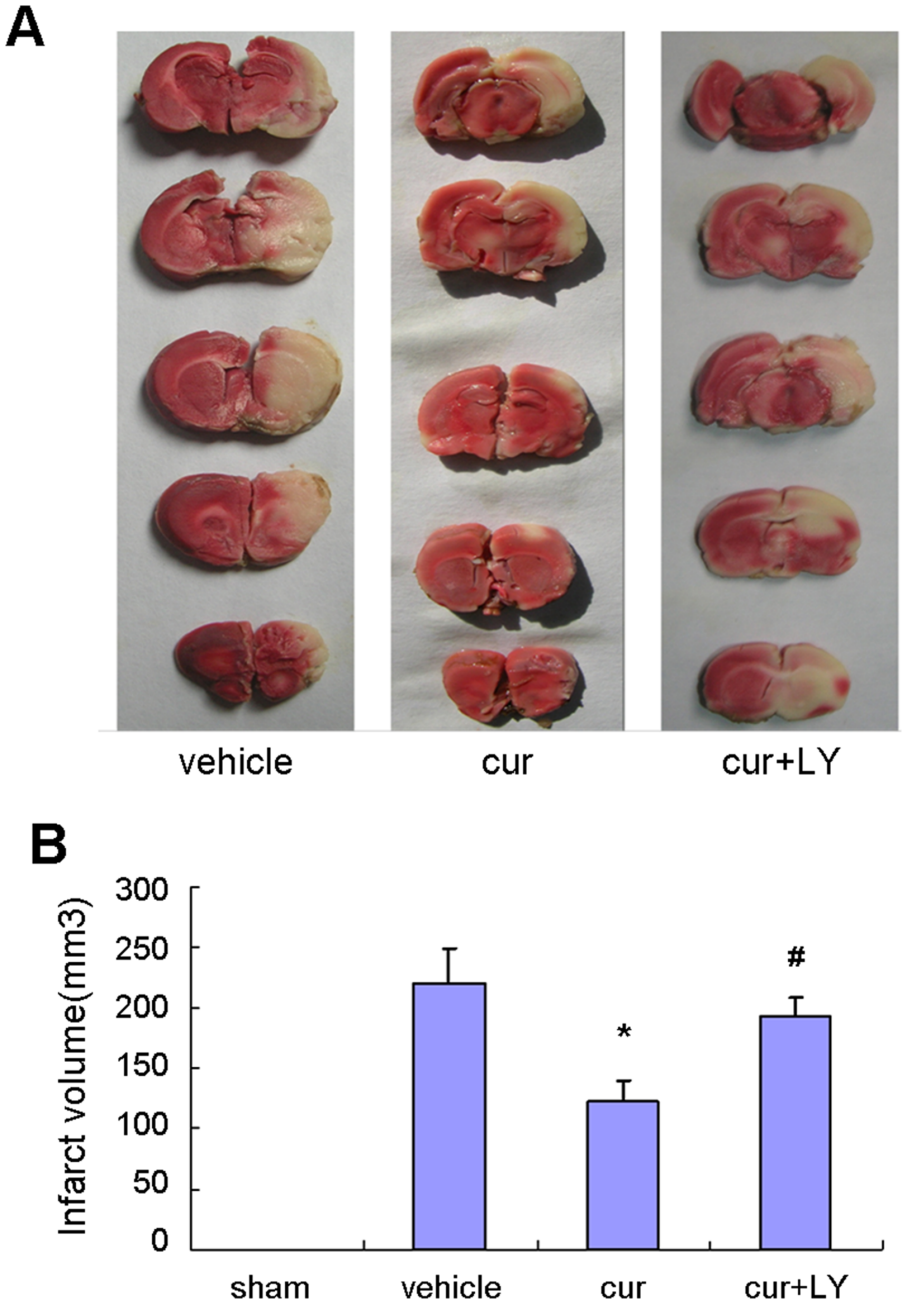
Curcumin attenuated focal cerebral I/R injury. A, TTC staining of representative coronal sections 24 hour after focal I/R. B, Quantification of infarct volumes 24 hour after focal ischemia. Bars represent mean±SEM (n = 8 for each group). *p<0.05 vs vehicle, #*p*<0.05 vs curcumin.

Curcumin significantly decreased infarct volumes 24 hours after reperfusion (Figure 3). As shown in Figure 3B, a remarkably decreased pale-colored region was observed in the curcumin treated rat compared with the vehicle group and cur+LY group. It is notable that the neuroprotective effects of curcumin to attenuate infarction volume were inhibited after combination with LY294002.

### Curcumin-Attenuated Oxidative Stress

**Figure 4:**
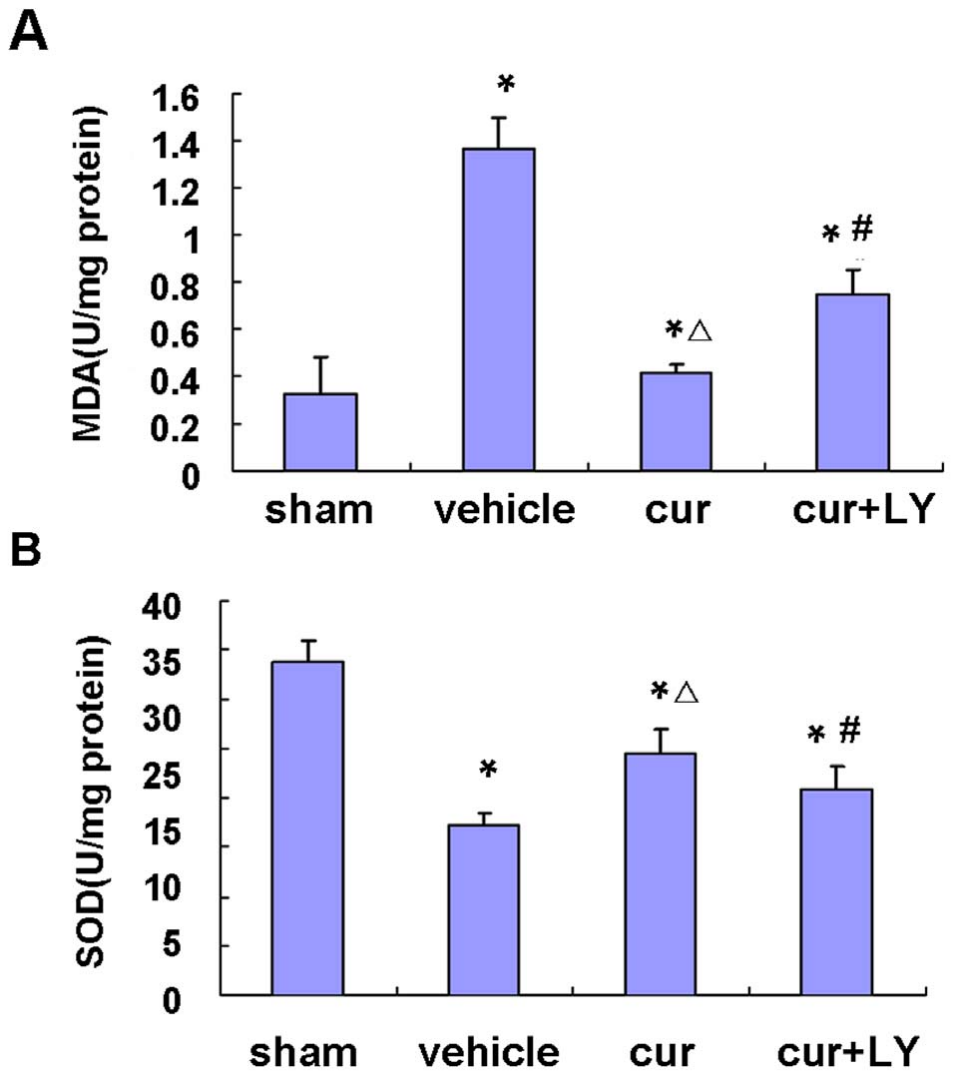
Effect of curcumin on oxidative stress. A, Assay of MDA content in the cortex. B, Assay of SOD activity in the cortex. Bars represent mean±SEM (n = 6–7 for each group). *p<0.05 vs sham, △*p*<0.05 vs vehicle and #*p*<0.05 vs curcumin.

The MDA level in the cortex, which is an index of lipid peroxidation, was significantly higher in the I/R group compared with the sham-operated group. There was significant reduction in the MDA level in the cur group compared with the vehicle group (Figure 4A). SOD activity in the cortex was decreased significantly in the vehicle group compared with the sham-operated group, which was significantly restored by curcumin (Figure 4B). In contrast, the combined use of LY294002 blocked the decrease of MDA level and the increase of SOD level induced by pretreatment with only curcumin.

### Curcumin-induced Nrf2-binding Activity to ARE

**Figure 5:**
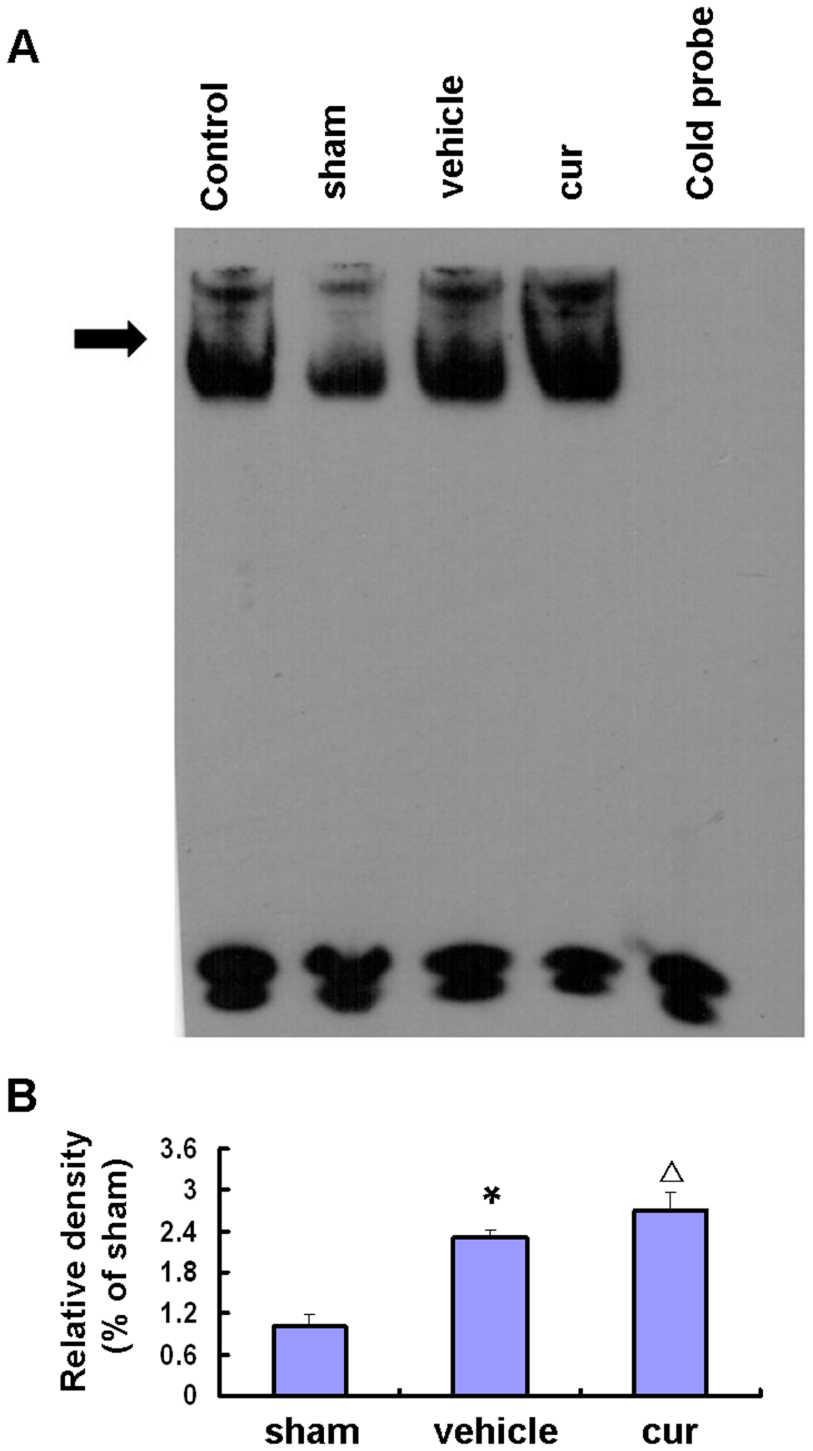
Curcumin-induced Nrf2-binding activity to ARE. A, Nrf2-binding activity to ARE: EMSA using the ARE probe in nuclear extracts of cortical rat neurons. The ARE-protein binding complexes (arrow) are indicated. B, semiquantitative analyses of Nrf2-binding activity to ARE. Bars represent mean±SEM (n = 6–8 for each group). *p<0.05 vs sham, △p<0.05 vs vehicle.

EMSA revealed that Nrf2-binding proteins were detected and low Nrf2-binding activity was observed in sham-operated rats (Figure 5, arrow-head). At 24 h after focal cerebral I/R, the Nrf2 activity was significantly increased in the vehicle group compared to the sham group (P<0.01), and the Nrf2 activity was markedly up-regulated in the curcumin group (P<0.05). Curcumin promoted Nrf2-DNA binding activities.

### PI3-kinase Inhibitor Blocked Phosphorylation of Akt and Suppressed Expression of Nrf2 and NQO1 Induced by Curcumin after MCAO

**Figure 6:**
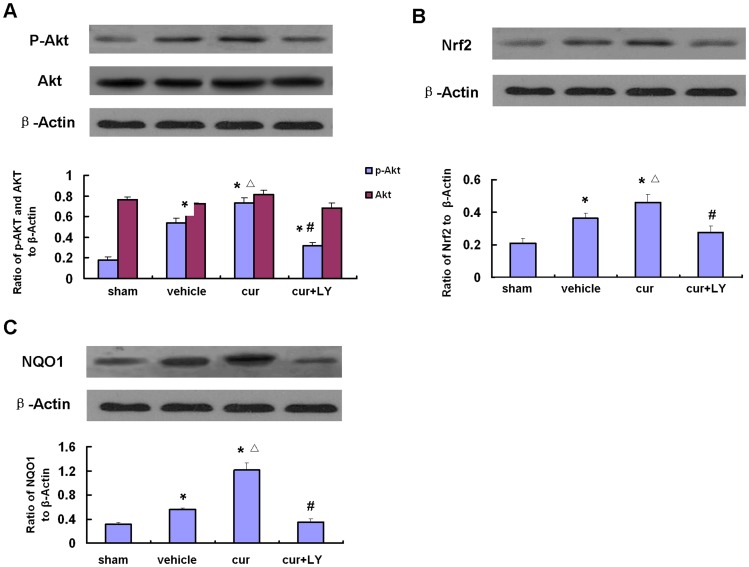
Western blot analysis of Akt, phospho-Akt, Nrf2 and NQO1 in the rat cortex after focal cerebral ischemia. A, Representative Western blots and semiquantitative analyses of phospho-Akt and Akt levels in MCA cortical tissues at 24 h. B, Representative Western blots and semiquantitative analyses of Nrf2 levels in MCA cortical tissues at 24 h. C, Representative Western blots and semiquantitative analyses of NQO1 levels in MCA cortical tissues at 24 h. Bars represent mean±SEM (n = 6–8 for each group). *p<0.05 vs sham, △*p*<0.05 vs vehicle and #*p*<0.05 vs curcumin.

Western blot analysis showed a significant increase in phospho-Akt, Nrf2 and NQO1 in the curcumin group compared to the vehicle and sham-operated groups, whereas the combined use of both curcumin and LY294002 blocked the increase of the Ser-473 phosphorylation of Akt, Nrf2 and NQO1 induced by post-treatment with only curcumin. Meanwhile, the protein levels of Akt were unaffected (Figure 6). Consistent with the western blot analysis, curcumin strongly increased expression levels of phospho-Akt, Nrf2 and NQO1 in the ischemic areas of the cortex. The increased expression of these proteins was inhibited in the curcumin- and LY group 24 h after MCAO (Figure 6).

## Discussion

Our study showed that curcumin improves survival of cortical neurons induced by OGD/R and reduced OGD-induced cell injury in vitro. In addition, our results revealed that curcumin decreases infarct volume and inhibits oxidative stress after focal cerebral ischemia/reperfusion injury in MCAO rats. Furthermore, we demonstrated for the first time that the PI3K/Akt signaling pathway plays a critical role in curcumin-mediated neuroprotection in both a rat model of MCAO and cortical neurons exposed to OGD, and the Nrf2 may also be involved.

Curcumin, a known antioxidant, has been reported to inhibit lipid peroxidation and to scavenge superoxide anions and hydroxyl radicals effectively [27]. Moreover, curcumin has been shown to activate a xenobiotic response in the cell by affecting the expression of detoxifying enzymes such as NQO1, GST, AR and HO-1 [28], [29]. In this study, LDH release was markedly decreased and accompanied by an increased cell survival, when curcumin was applied to neurons exposed to OGD/R. In addition, our results indicated that a rapid increase in the intracellular expression of NQO1 induced by OGD was enhened by curcumin post-treatment, which paralleled with attenuated cell injury. Our present study has also demonstrated that curcumin decreases the level of MDA product, increases SOD activity and the expression of NQO1 after MCAO. NQO1 is generally considered a detoxifying enzyme, because of its ability to reduce reactive quinones and quinone imines to less reactive and less toxic hydroquinones [30]. NQO1 is highly inducible by many stimuli including electrophilic metabolites and oxidative stress, and its induction is considered to be transcriptionally regulated by ARE [31], [32]. In this study, we demonstrated that after 24 h reoxygenation with curcumin cultured cortical neurons increased the transcription level of NQO1 and the addition of LY294002 (signaling inhibitors for PI3K) reduced the NQO1 expression. These data indicate that up-regulation NQO1 expression in neurons by curcumin requires activation of the PI3-K/Akt pathway in vitro.

Although the antioxidative molecular mechanism of curcumin is unclear, recent studies have found that curcumin induces the expression of HO-1 [33] and AR [18] in vitro through a PI3K/Akt-mediated signaling pathway involving the transcription factor Nrf2. To verify whether curcumin inhibited oxidative stress and induced the expression of NQO1 after focal cerebral ischemia/reperfusion injury in rats via PI3K/Akt pathway involving Nrf2, we performed a PI3K/Akt inhibition study with LY294002. To identify whether curcumin induced the binding of Nrf2 to ARE in MCAO rats, we applied EMSA to assess Nrf2-binding activity. Our findings supported an important role for the PI3K/Akt pathway involving Nrf2 in curcumin-mediated neuroprotection against ischemic neuronal injury. The phosphorylation of Akt was increased quickly and shortly after ischemia and reperfusion [34]. The short-term phosphorylation of Akt is also an essential component in inducing neuroprotection by other well-characterized mechanisms, as p-Akt levels are enhanced after a variety of protective agents, including insulin-like growth factor-1 [10], erythropoietin [35], and estrogen [36]. Akt was necessary for the neuroprotective effects of the aforementioned agents, because blocking the PI3K/Akt signaling pathway abolished their neuroprotective effects.

The neuroprotective mechanisms of Akt may involve activation of a set of transcription factors, including Nrf2, which is considered to be a multi-organ protector and is widely viewed as a mediator of neuroprotection by up-regulating the expression of many detoxifying and antioxidant enzymes under oxidative stress [37]. Nrf2 is the major transcription factor that binds to and activates the expression of these ARE-mediated gene products [38]. It has been reported that multiple signaling pathways mediate the induction of Nrf2-driven phase II enzymes including PI3K/Akt, MEK/ERK, p38MAPK, JNK, and protein kinase C [39]. We observed remarkable up-regulation of phospho-Akt and NQO1 in MCAO rats following treatment with curcumin (5 µM), and this up-regulation was accompanied by increases in the nuclear translocation of Nrf2 and in the DNA-binding of Nrf2 to the ARE sequence. In the present study, we reported that the cerebral Nrf2 activity was significantly activated and could be induced by curcumin in the rats MCAO model. These results provide evidence that activation of Nrf2 by administration of curcumin is a potential mechanism for its neuroprotection after MCAO.

The present study has demonstrated up-regulation of NQO1 expression mediated by PI3K/Akt and showed that Nrf2 is involved in the neuroprotective effects of curcumin against oxidative damage at a relevant time point. Further studies should investigate whether other signaling pathways also involve the antioxidative effects of curcumin. Moreover, because curcumin has multiple therapeutic properties such as antioxidative anti-inflammatory properties and so on, we should consider if neuroprotection of curcumin may involve anti-inflammation.

### Conclusions

Curcumin, which is safe and has low toxicity, has been used for centuries in indigenous medicines for the treatment of a variety of medical conditions [40]. In our study, curcumin was effective in promoting neuronal survival after OGD/R and reducing experimental ischemic injury induced by MCAO in rats. Taken together, this suggests that curcumin may be useful in treating ischemic events in humans. Further studies will be needed to clarify the mechanisms involved.

## References

[1] Lim GP, Chu T, Yang F, Beech W, Frautschy SA, et al (2001) The curry spice curcumin reduces oxidative damage and amyloid pathology in an Alzheimer transgenic mouse. J Neurosci 21: 8370–8377. 11606625 10.1523/JNEUROSCI.21-21-08370.2001 PMC6762797

[2] Dutta S, Padhye S, Priyadarsini KI, Newton C (2005) Antioxidant and antiproliferative activity of curcumin semicarbazone. Bioorg Med Chem Lett 15: 2738–2744. 15878268 10.1016/j.bmcl.2005.04.001

[3] Biswas SK, McClure D, Jimenez LA, Megson IL, Rahman I (2005) Curcumin induces glutathione biosynthesis and inhibits NF-kappaB activation and interleukin-8 release in alveolar epithelial cells: mechanism of free radical scavenging activity. Antioxid Redox Signal 7: 32–41. 15650394 10.1089/ars.2005.7.32

[4] Singh S, Khar A (2006) Biological effects of curcumin and its role in cancer chemoprevention and therapy. Anticancer Agents Med Chem 6: 259–270. 16712454 10.2174/187152006776930918

[5] Ghoneim AI, Abdel-Naim AB, Khalifa AE, El-Denshary ES (2002) Protective effects of curcumin against ischaemia/reperfusion insult in rat forebrain. Pharmacol Res 46: 273–279. 12220971 10.1016/s1043-6618(02)00123-8

[6] Thiyagarajan M, Sharma SS (2004) Neuroprotective effect of curcumin in middle cerebral artery occlusion induced focal cerebral ischemia in rats. Life Sci 74: 969–985. 14672754 10.1016/j.lfs.2003.06.042

[7] Wang Q, Sun AY, Simonyi A, Jensen MD, Shelat PB, et al (2005) Neuroprotective mechanisms of curcumin against cerebral ischemia-induced neuronal apoptosis and behavioral deficits. J Neurosci Res 82: 138–148. 16075466 10.1002/jnr.20610

[8] Zhao J, Zhao Y, Zheng W, Lu Y, Feng G, et al (2008) Neuroprotective effect of curcumin on transient focal cerebral ischemia in rats. Brain Res 1229: 224–232. 18640105 10.1016/j.brainres.2008.06.117

[9] Zhao J, Yu S, Zheng W, Feng G, Luo G, et al (2010) Curcumin improves outcomes and attenuates focal cerebral ischemic injury via antiapoptotic mechanisms in rats. Neurochem Res 35: 374–379. 19774461 10.1007/s11064-009-0065-y

[10] Kulik G, Klippel A, Weber MJ (1997) Antiapoptotic signalling by the insulin-like growth factor I receptor, phosphatidylinositol 3-kinase, and Akt. Mol Cell Biol 17: 1595–1606. 9032287 10.1128/mcb.17.3.1595 PMC231885

[11] Vaillant AR, Mazzoni I, Tudan C, Boudreau M, Kaplan DR, et al (1999) Depolarization and neurotrophins converge on the phosphatidylinositol 3-kinase-Akt pathway to synergistically regulate neuronal survival. J Cell Biol 146: 955–966. 10477751 10.1083/jcb.146.5.955 PMC2169479

[12] Williams EJ, Doherty P (1999) Evidence for and against a pivotal role of PI 3-kinase in a neuronal cell survival pathway. Mol Cell Neurosci 13: 272–280. 10328886 10.1006/mcne.1999.0750

[13] Benson AM, Hunkeler MJ, Talalay P (1980) Increase of NAD(P)H:quinone reductase by dietary antioxidants: possible role in protection against carcinogenesis and toxicity. Proc Natl Acad Sci U S A 77: 5216–5220. 6933553 10.1073/pnas.77.9.5216 PMC350028

[14] Yang FY, Guan QK, Cui YH, Zhao ZQ, Rao W, et al.. (2012) NAD(P)H quinone oxidoreductase 1 (NQO1) genetic C609T polymorphism is associated with the risk of digestive tract cancer: a meta-analysis based on 21 case-control studies. Eur J Cancer Prev. 10.1097/CEJ.0b013e32834f7514 22387672

[15] Benson AM, Batzinger RP, Ou SY, Bueding E, Cha YN, et al (1978) Elevation of hepatic glutathione S-transferase activities and protection against mutagenic metabolites of benzo(a)pyrene by dietary antioxidants. Cancer Res 38: 4486–4495. 363262

[16] Benson AM, Cha YN, Bueding E, Heine HS, Talalay P (1979) Elevation of extrahepatic glutathione S-transferase and epoxide hydratase activities by 2(3)-tert-butyl-4-hydroxyanisole. Cancer Res 39: 2971–2977. 455282

[17] Alam J, Camhi S, Choi AM (1995) Identification of a second region upstream of the mouse heme oxygenase-1 gene that functions as a basal level and inducer-dependent transcription enhancer. J Biol Chem 270: 11977–11984. 7538129 10.1074/jbc.270.20.11977

[18] Kang ES, Woo IS, Kim HJ, Eun SY, Paek KS, et al (2007) Up-regulation of aldose reductase expression mediated by phosphatidylinositol 3-kinase/Akt and Nrf2 is involved in the protective effect of curcumin against oxidative damage. Free Radic Biol Med 43: 535–545. 17640564 10.1016/j.freeradbiomed.2007.05.006

[19] Ming Y, Zhang H, Long L, Wang F, Chen J, et al (2006) Modulation of Ca2+ signals by phosphatidylinositol-linked novel D1 dopamine receptor in hippocampal neurons. J Neurochem 98: 1316–1323. 16771826 10.1111/j.1471-4159.2006.03961.x

[20] Wu X, Zhao J, Yu S, Chen Y, Wu J, et al (2012) Sulforaphane protects primary cultures of cortical neurons against injury induced by oxygen-glucose deprivation/reoxygenation via antiapoptosis. Neurosci Bull 28: 509–516. 23054633 10.1007/s12264-012-1273-z PMC5561925

[21] Chen Y, Wu X, Yu S, Lin X, Wu J, et al (2012) Neuroprotection of Tanshinone IIA against Cerebral Ischemia/Reperfusion Injury through Inhibition of Macrophage Migration Inhibitory Factor in Rats. PLoS One 7: e40165. 22768247 10.1371/journal.pone.0040165 PMC3387137

[22] Yu SS, Zhao J, Zheng WP, Zhao Y (2010) Neuroprotective effect of 4-hydroxybenzyl alcohol against transient focal cerebral ischemia via anti-apoptosis in rats. Brain Res 1308: 167–175. 19857470 10.1016/j.brainres.2009.10.037

[23] Berti R, Williams AJ, Moffett JR, Hale SL, Velarde LC, et al (2002) Quantitative real-time RT-PCR analysis of inflammatory gene expression associated with ischemia-reperfusion brain injury. J Cereb Blood Flow Metab 22: 1068–1079. 12218412 10.1097/00004647-200209000-00004

[24] Wei X, Liu H, Sun X, Fu F, Zhang X, et al (2005) Hydroxysafflor yellow A protects rat brains against ischemia-reperfusion injury by antioxidant action. Neurosci Lett 386: 58–62. 16002219 10.1016/j.neulet.2005.05.069

[25] Zhou ML, Zhu L, Wang J, Hang CH, Shi JX (2007) The inflammation in the gut after experimental subarachnoid hemorrhage. J Surg Res 137: 103–108. 17069855 10.1016/j.jss.2006.06.023

[26] Alam J, Cook JL (2003) Transcriptional regulation of the heme oxygenase-1 gene via the stress response element pathway. Curr Pharm Des 9: 2499–2511. 14529549 10.2174/1381612033453730

[27] Kalpana C, Sudheer AR, Rajasekharan KN, Menon VP (2007) Comparative effects of curcumin and its synthetic analogue on tissue lipid peroxidation and antioxidant status during nicotine-induced toxicity. Singapore Med J 48: 124–130. 17304391

[28] Balamurugan AN, Akhov L, Selvaraj G, Pugazhenthi S (2009) Induction of antioxidant enzymes by curcumin and its analogues in human islets: implications in transplantation. Pancreas 38: 454–460. 19188863 10.1097/MPA.0b013e318196c3e7

[29] Van Erk MJ, Teuling E, Staal YC, Huybers S, Van Bladeren PJ, et al (2004) Time- and dose-dependent effects of curcumin on gene expression in human colon cancer cells. J Carcinog 3: 8. 15140256 10.1186/1477-3163-3-8 PMC421747

[30] Lind C, Cadenas E, Hochstein P, Ernster L (1990) DT-diaphorase: purification, properties, and function. Methods Enzymol 186: 287–301. 2233301 10.1016/0076-6879(90)86122-c

[31] Jaiswal AK (2000) Regulation of genes encoding NAD(P)H:quinone oxidoreductases. Free Radic Biol Med 29: 254–262. 11035254 10.1016/s0891-5849(00)00306-3

[32] Dinkova-Kostova AT, Talalay P (2010) NAD(P)H:quinone acceptor oxidoreductase 1 (NQO1), a multifunctional antioxidant enzyme and exceptionally versatile cytoprotector. Arch Biochem Biophys 501: 116–123. 20361926 10.1016/j.abb.2010.03.019 PMC2930038

[33] Pugazhenthi S, Akhov L, Selvaraj G, Wang M, Alam J (2007) Regulation of heme oxygenase-1 expression by demethoxy curcuminoids through Nrf2 by a PI3-kinase/Akt-mediated pathway in mouse beta-cells. Am J Physiol Endocrinol Metab 293: E645–655. 17535857 10.1152/ajpendo.00111.2007

[34] Noshita N, Lewén A, Sugawara T, Chan PH (2001) Evidence of phosphorylation of Akt and neuronal survival after transient focal cerebral ischemia in mice. J Cereb Blood Flow Metab 21: 1442–1450. 11740206 10.1097/00004647-200112000-00009

[35] Kilic E, Kilic U, Soliz J, Bassetti CL, Gassmann M, et al (2005) Brain-derived erythropoietin protects from focal cerebral ischemia by dual activation of ERK-1/−2 and Akt pathways. FASEB J 19: 2026–2028. 16207820 10.1096/fj.05-3941fje

[36] Yune TY, Park HG, Lee JY, Oh TH (2008) Estrogen-induced Bcl-2 expression after spinal cord injury is mediated through phosphoinositide-3-kinase/Akt-dependent CREB activation. J Neurotrauma 25: 1121–1131. 18785877 10.1089/neu.2008.0544

[37] Enomoto A, Itoh K, Nagayoshi E, Haruta J, Kimura T, et al (2001) High sensitivity of Nrf2 knockout mice to acetaminophen hepatotoxicity associated with decreased expression of ARE-regulated drug metabolizing enzymes and antioxidant genes. Toxicol Sci 59: 169–177. 11134556 10.1093/toxsci/59.1.169

[38] Narasimhan M, Mahimainathan L, Rathinam ML, Riar AK, Henderson GI (2011) Overexpression of Nrf2 protects cerebral cortical neurons from ethanol-induced apoptotic death. Mol Pharmacol 80: 988–999. 21873460 10.1124/mol.111.073262 PMC3228534

[39] Lee JS, Surh YJ (2005) Nrf2 as a novel molecular target for chemoprevention. Cancer Lett 224: 171–184. 15914268 10.1016/j.canlet.2004.09.042

[40] Lodha R, Bagga A (2000) Traditional Indian systems of medicine. Ann Acad Med Singapore 29: 37–41. 10748962

